# Mortality and hospital admissions in people with eating disorders. A longitudinal cohort study in secondary-care linked English primary care records

**DOI:** 10.1192/bjp.2025.69

**Published:** 2025-06-23

**Authors:** Annie Jeffery, Joseph F. Hayes, Naomi Launders, Glyn Lewis, David Osborn, Helen Bould, Naomi Warne, Francesca Solmi

**Affiliations:** 1 Division of Psychiatry, UCL, London, UK; 2 North London Mental Health Partnership, London, UK; 3 Centre for Academic Mental Health, Population Health Sciences, Bristol Medical School, University of Bristol; 4 Gloucestershire Health and Care NHS Foundation Trust, Gloucestershire, United Kingdom; 5 MRC Integrative Epidemiology Unit, Bristol Medical School, University of Bristol, United Kingdom

## Abstract

**Background:**

Research on mortality and admissions for physical health problems across eating disorder diagnoses in representative settings is scarce. Inequalities in these outcomes across a range of socio-demographic characteristics have rarely been investigated.

**Aims:**

We investigated whether people with eating disorders had greater all-cause mortality and physical health-related inpatient admissions compared to people without eating disorders and whether associations varied by sex, ethnicity, deprivation, age, and calendar year at diagnosis.

**Methods:**

Using primary care Clinical Research Practice Datalink linked to Hospital Episode Statistics, we matched people with an incident eating disorder diagnosis (any, anorexia nervosa, bulimia nervosa, EDNOS, generic eating disorder, or a referral code) from primary care Read codes to four people without eating disorders (1:4 matching) on year of birth, sex, primary care practice, year of registration, and index date. We used univariable and multivariable Cox (mortality) and Poisson (admissions) models and fitted interactions to investigate whether associations varied by socio-demographic characteristics.

**Results:**

We included 58,735 people (90.1% female, 91.6% white). People with any eating disorders had higher all-cause mortality (Hazard Ratio[HR]: 2.15, 95% confidence interval [CI]: 1.73 – 2.67). Anorexia nervosa had the highest mortality (HR: 3.49, 95%CI: 2.43 – 5.01). People with any eating disorders had higher rates of planned (Incidence Rate Ratio[IRR]: 1.80, 95%CI: 1.4 – 1.87) and emergency admissions for physical health problems (IRR: 2.35. 95%CI: 2.35 – 2.46) and emergency admissions for injuries, accidents and substance misuse (IRR: 5.26, 95%CI: 5.24 – 5.29). Mortality and admissions rate ratios were greater in males.

**Conclusions:**

People with eating disorders have high rates of mortality and physical health-related admissions. Observed inequalities call for an understanding of why such inequalities exist. These findings highlight the need for prompt and effective treatment for eating disorders and for improved guidance on primary care management of people with eating disorders.

## Introduction

People being treated for eating disorders have an increased risk of mortality compared with the general population. The highest mortality rates are observed in people with anorexia nervosa(1–11), however, they appear to be also elevated in people with bulimia nervosa and eating disorders not otherwise specified (EDNOS)(1,5–7,10,12,13), albeit research is more limited for these conditions. There is also evidence that people with eating disorders have higher secondary healthcare usage(11,14–17) and expenditure (18) compared to the general population, but only a handful of studies have focused on physical health admissions. These found elevated risk of the latter in young people with any eating disorders(15) and people with binge eating disorder(17), and of cardiovascular admissions in women with bulimia nervosa(16). This body of research highlights the potential clinical severity and physical health problems associated with eating disorders but has a number of limitations. With some exceptions(10,11,19), existing studies of eating disorder mortality have been based on relatively small samples of people with eating disorders attending specialised eating disorder services; this could bias estimates if this is a sub-group of patients with more severe symptoms, while also reducing statistical power. When studies have used primary care records to investigate mortality, they have not disaggregated estimates by eating disorder diagnosis(11) or have not accounted for possible socio-demographic confounders, including ethnicity or deprivation.(10) Most research on healthcare utilisation in people with eating disorders has been based in North America (14–16), has only focused on specific conditions(16–18) or populations(15,16), did not disaggregate admissions by type (e.g., planned or emergency), and had relatively small sample sizes and short follow up times.(14,15) With few exceptions, studies have also defined people with eating disorders as those receiving diagnoses in secondary settings(14–18) but, at times, used general population controls, which could bias results.(15) Although several studies have investigated predictors of mortality(2), admissions(20), and healthcare costs(21) within eating disorder populations, inequalities in these outcomes in people with eating disorders compared to the general population have been sparsely investigated. A handful of studies investigated sex differences in mortality with mixed findings; some found no differences (5,10), others observed higher mortality in women(9,10), and, still, others noted higher mortality in males.(2) A recent Australian study has investigated socioeconomic inequalities in hospital admissions in people with eating disorders finding greater public outpatient and emergency admissions in those from more deprived socioeconomic backgrounds, but no other differences.(22) To our knowledge, there is no evidence on whether there are ethnic differences in mortality and admissions between people with eating disorders and the general population.

### Study aims

To address these limitations, we used secondary care-linked English primary care electronic health records to investigate whether people with any and specific eating disorder diagnoses have increased mortality rates. In England, only a portion of primary care patients receive a referral to specialised services(19) making primary care a more representative sample of all people with eating disorder compared to those presenting to eating disorder services. In this dataset, we also investigated whether people with eating disorders have increased rates of hospital admissions for physical health problems (any admissions, planned and emergency admissions, and emergency admissions for accidents, injuries, and substance misuse) compared to people without eating disorders. Finally, we investigated whether relative rates of mortality and admissions differed by sex, ethnicity, deprivation, age, and calendar year. These have not been consistently and robustly explored in the previous literature, despite evidence of potential barriers in seeking and receiving treatment in males and people from deprived or minoritized ethnic backgrounds (23,24) which could translate in worse outcomes for these populations.

## Methods

### Sample

We used data from the UK Clinical Practice Research Datalink (CPRD Gold and Aurum, constituting a subset of all English primary care practices) linked to Hospital Episode Statistics (HES) Admitted Patient Care dataset using pseudonymised unique patient identifiers (Supplemental method 1). The Independent Scientific Advisory Committee of CPRD approved this study (protocol no. 18_288).

In the main analytical sample, we included people who were registered at a CPRD primary care practice in England with HES linkage between 1^st^ January 2000 to 31^st^ December 2018 and who had at least one clinical code for an eating disorder (list in Supplemental method 2) recorded between the ages of 11 to 60, and between 1^st^ January 2000 to 31^st^ December 2017 to allow at least one year of potential follow-up. Each person was matched to up to four patients without any record of an eating disorder. The matching was performed by the CPRD based on year of birth, sex, primary care practice, year of GP registration and index date. All individuals had to have at least one year of follow up data after diagnosis date. Main analyses were restricted to people included in CPRD who had records linked to HES to ensure sample comparability between people included in analyses of mortality and admissions (derived from HES linkage). As sensitivity analyses, we repeated the mortality analyses in the full primary care dataset (i.e. including Wales, Scotland and Northern Ireland) to increase sample size.

### Eating disorder diagnoses

The primary exposure was whether an individual had any eating disorder diagnosis vs none. As a secondary exposure, we compared individual eating disorder diagnoses with not having an eating disorder diagnosis. Records of eating disorder diagnoses were derived from primary care Read codes and included the following categories: anorexia nervosa, bulimia nervosa, and EDNOS (more details in Supplemental method 3). In addition to these diagnoses, there were two groups of people, one with a generic ‘eating disorder’ code and one with a code indicating that a ‘referral to an eating disorder service’ had been made, but who were never subsequently given a more specific diagnostic code. We kept these two groups separate as we were not able to link their codes to a specific diagnosis.

### Outcome-Mortality and hospital admissions for physical health problems

Primary study outcomes were all-cause mortality (defined using date of death recorded in primary care records) and any inpatient hospital admission (defined as a spell of continuous hospitalisation in a single hospital) for physical health problems defined using ICD10 diagnoses as primary diagnoses. Secondary study outcomes were planned and emergency admissions for physical health problems, and emergency admissions for accidents, injuries, and substance misuse. We identified secondary care admissions through linkage with the admitted patient care HES dataset (Supplemental Method 4). We excluded admissions for maternity, regular attenders for the same condition, e.g. cancer treatment, and any admissions resulting from a transfer from another hospital, including mental health settings.

### Confounders

We included age at eating disorder diagnosis, sex (male/female), ethnicity (categorised as Asian, Black, Mixed, Other and White), deprivation captured using the Index of Multiple Deprivation (IMD, definition in supplemental method 5) associated with individuals’ home address and split in fifths of distribution, calendar year of eating disorder diagnosis, as potential confounders of the associations under study. The only variable with missing data was ethnicity. Where ethnicity was missing, we recoded this as ‘White’. As the CPRD population has been found to be representative of the UK population in terms of ethnicity(25), 93% or more individuals with missing ethnicity would be expected to be of White ethnicity. This approach is in line with other research studies using CPRD data(26) and findings of studies showing that their results were comparable when using this approach or using multiple imputation to impute missing ethnicity data.(27)

### Data analysis

In reporting results, we followed STROBE guidelines (Supplemental method 6). We described the sample using frequencies and proportions. To investigate whether those with any or a specific eating disorder diagnosis were at increased risk of all-cause mortality we used Cox regression models, after confirming that the proportionality of hazards assumption was met. To investigate whether people with any or specific eating disorder diagnoses had a higher incidence of admissions we used Poisson regression models. In both sets of analyses, we first ran a univariable analysis, and then a multivariable analysis, adjusting for patient’s sex, ethnicity, age, deprivation, region, and calendar year, clustering analyses by primary care practice. Participants were followed up from the day they received an eating disorder diagnosis (the same date was used in unexposed patients) until they died, changed practice, or the end of the study, whichever occurred first.

We further investigated whether patients with and without eating disorders had differential mortality and admission rates based on sex, ethnicity, age, deprivation and calendar year by testing for an interaction between each of these variables and the exposure in multivariable models. In these analyses we grouped all eating disorder subtypes in order to increase statistical power. Finally, for admission analyses, we also presented crude admission rates by specific condition based on broad ICD10 code classification.

As sensitivity analyses, to increase statistical power, we re-ran analyses of all-cause mortality on the full CPRD cohort of patients, i.e., without restricting to participants with HES linkage. Here, we did not adjust for IMD, as the latter is only available on the subset of patients with linked data. All analyses were conducted in R Version 4.2.3. Ethical approval for this study was obtained from the Independent Scientific Advisory Committee of CPRD.

## Results

### Sample

A total of 33,526 people aged 11 to 60 years received an eating disorder diagnosis in a CPRD-registered primary care practice between 1^st^ January 2000 and 31^st^ December 2017. After matching these individuals to four people without eating disorders, we obtained a sample of 167,630 people, which we used in sensitivity analyses of all-cause mortality. Of these people, 58,735 (35.0%:12,129 with and 46,606 without an eating disorder diagnosis) had linked HES records and were therefore included in our main analytical sample.

The majority of the sample was female (n=52,949, 90.1%), of white ethnicity (n=53,819, 91.6%) and under 30 years of age (n=44,260, 75.3%). A greater proportion of people lived in the least deprived (n=13,402, 22.8%) compared to most deprived areas of England (n=8,973, 15.3%). The distribution of age and sex did not differ in those with and without eating disorders, as individuals were matched on these characteristics. However, there were a greater proportion of white people (94.6% vs 90.8%) and fewer people living in the most deprived areas (21.8% vs 23.1%) in those with an eating disorder than in those without, respectively (Table 1).

Among those with eating disorders, the most common diagnosis was EDNOS (n=3,542, 29.2%), followed by a generic eating disorder code (n=2,851, 23.5%), a diagnosis of anorexia nervosa (n=2,513, 20.8%), a diagnosis of bulimia nervosa (n=2,186, 18.0%), and only a referral code (n=1,037, 8.5%). Fewer men and people from ethnic minority backgrounds and greater proportions of people aged 11-20 years were diagnosed with anorexia nervosa and bulimia nervosa compared to EDNOS and generic eating disorder diagnoses (Table 1).

### All-cause Mortality

People with any eating disorder diagnoses had higher rates of all-cause mortality (multivariable hazard ratio [mHR]: 2.15, 95% confidence interval [CI]: 1.73 – 2.67). People with anorexia nervosa had the most elevated hazard ratios for all-cause mortality (mHR: 3.49, 95%CI: 2.43 – 5.01); these were also elevated for those with EDNOS (mHR: 2.11, 95%CI: 1.54 – 2.90) and a generic eating disorder diagnosis (mHR: 2.14, 95%CI: 1.47 – 3.12). There was no evidence that mortality rates were elevated in people with bulimia nervosa (mHR: 1.20, 95%CI: 0.84 – 2.08) and in those with a single referral code (mHR: 1.27, 95%CI: 0.56 – 2.86, Table 2). Proportional hazards assumptions were met (Schoenfeld p value=0.87 for main analyses, and p=0.73 for diagnosis-specific analyses) suggesting that increased risk of mortality observed was constant across the follow up period.

There was strong evidence that mortality hazard ratios were more elevated in males (mHR: 4.60, 95%CI: 2.74 – 7.73) than in females (mHR: 1.85, 95%CI: 1.45-2.35) with any eating disorder (interaction p = 0.0038). Evidence of differences by other socio-demographic characteristics was weak for deprivation (interaction p-value=0.05, least deprived IMD fifth HR: 2.12, 95%CI: 1.23 – 3.64; most deprived IMD fifth HR: 3.17, 95% CI: 2.02-4.99) and ethnicity (p=0.09; White ethnicity HR: 2.10, 95% CI: 1.69 – 2.62; ethnic minority HR: 3.91, 95% CI: 1.30-11.75). There was no evidence of other interactions (Table 3).

### Physical Health Admissions

#### Any physical health admissions

There was strong evidence that people with eating disorders had higher incidence of any physical health hospital admissions (multivariable Incidence Rate Ratio [mIRR]: 1.99, 95%CI: 1.94 – 2.05). Rate ratios were most elevated in patients with anorexia nervosa(mIRR: 2.28, 95% CI: 2.17 – 2.40) and lowest in those with a generic eating disorder code (mIRR: 1.62, 95%CI: 1.54 – 1.71, Table 4).

#### Cause-specific admissions

There was strong evidence that people with eating disorders had elevated rates of admissions across all physical health-related causes. Rate ratios were most elevated for admissions for accidents, injuries, and substance misuse (mIRR: 5.26, 95%CI: 5.24 – 6.29), followed by emergency admissions (mIRR: 1.67. 95%CI: 1.62 – 1.72), and planned admissions (mIRR: 2.35, 95%CI: 2.25 – 2.46). Overall, we observed this pattern across all diagnoses (Table 4).

Patients with anorexia nervosa (mIRR: 7.07, 95%CI: 6.20 – 8.05) and bulimia nervosa (mIRR: 6.42, 95%CI: 5.53 – 7.42) had the most elevated rate ratios for emergency admissions for accidents, injuries, and substance misuse, whereas other emergency admissions were most elevated for patients with anorexia nervosa (mIRR: 3.11, 95%CI: 2.89 – 3.34) and EDNOS (mIRR: 2.49, 95%CI: 2.32 – 2.66). Incidence rate ratios of planned admissions were highest for patients with EDNOS (mIRR: 2.16, 95%CI: 2.05 – 2.23, Table 4).

With the exception of ear disorders, where admissions rates were low or comparable between people with and without eating disorders, the former had higher rates of physical health admissions for all other ICD-10 broad categories of disorders. People with eating disorders had particularly elevated rates of endocrine disorders (especially those with anorexia nervosa), disorders of the digestive and genitourinary systems. People with bulimia nervosa had the highest rates of cardiovascular diseases and those with anorexia nervosa and EDNOS the highest rates of cancer (Supplemental Table 1).

#### Interactions with socio-demographic characteristics

There was evidence (interaction p=0.01) that the association between having any eating disorder and any physical health admission was more pronounced in males (mIRR: 2.28, 95% CI: 2.07 – 2.51) compared to females (mIRR: 1.97, 95%CI: 1.91 – 2.33). There was also weak evidence that this association varied by IMD (interaction p=0.06) – with some evidence of more elevated rate ratios in those living in the more deprived areas although 95% confidence intervals mostly overlapped (Table 5). There was no evidence of other interactions.

### Sensitivity analyses

When we repeated the all-cause mortality analyses in the full primary care sample regardless of secondary care linkages and IMD data availability (N=167,630), results were comparable to those of the main analyses, but 95% confidence intervals were more precise around the estimates (Tables 2, 3). In contrast to the main analyses, here we observed strong evidence of increased mortality rates in patients with bulimia nervosa (mHR: 1.42, 95%CI: 1.05-1.91) and in those who only ever received a referral code (mHR: 1.57, 95%CI: 1.05 – 2.37), compared to people without eating disorders.

## Discussion

In this large cohort study using primary care electronic health records linked to secondary care, we described patterns of mortality and physical health admission rates in people with eating disorders compared to a matched group of people without eating disorders. We found that people with anorexia nervosa had over three-fold rates of all-cause mortality compared with people without eating disorders, and that those with a generic eating disorder code, or EDNOS had up to two-fold increased rates. These findings from a population cohort corroborate previous literature based on samples of patients in secondary care settings showing elevated mortality rates in particular in those with anorexia nervosa.(1,5–7,12,13) Our study expands on these earlier studies by observing increased mortality in patients identified in primary care and across eating disorder diagnoses, including those considered as having sub-threshold levels of severity or lacked a distinct diagnosis.

As far as we are aware, this is the first study comparing patterns of admissions for physical health problems in people with and without eating disorders in England, overall and by diagnosis. We found that people with eating disorders had high rates of admissions, with progressively higher rate ratios observed for planned admissions, emergency admissions for physical health problems, and emergency accidents, injuries and substance misuse. We also observed several distinct patterns. Patients with anorexia nervosa and bulimia nervosa had the most elevated rate ratios of emergency accidents, injuries and substance misuse, which could denote episodes of self-harm or attempted suicide. Clinical and general population evidence suggests that self-harm is common across eating disorder diagnoses, and particularly in people with bulimia nervosa(28,29) and purging behaviours,(30) whereas a recent systematic review found that the twelve-month prevalence of substance use disorder is elevated in both bulimia nervosa (6.0%) and anorexia nervosa (12.0%).(31) Finally, albeit only in descriptive analyses, we observed associations with endocrine (particularly for anorexia nervosa), digestive, genitourinary, and cardiovascular (particularly for bulimia nervosa) conditions, which have all been previously described in this population(16,28–33). We also found a large incidence of cancer in patients with anorexia nervosa and EDNOS. Previous studies found lower incidence of some cancers (e.g. breast cancer) in anorexia nervosa, but higher incidence of others (e.g. lung or oesophageal) cancers in this population.(34)

When we investigated whether patterns of mortality and physical health admissions differed by socio-demographic characteristics, we observed some stark differences. Despite having lower incidence of eating disorders, compared to women, men had more elevated rate ratios of mortality and admissions, albeit differences in the latter were less pronounced. This finding could mean that men might have more severe eating disorder presentations at diagnosis and/or might be less able to access eating disorder services promptly. Although research is limited, both quantitative and qualitative studies converge in showing that men might face diagnostic delays in seeking and receiving treatment, potentially due to internalised stigma, and might be faced with largely female-based eating disorder-related health information provided when receiving care, making treatment less accessible.(35) Similarly, although in England the recorded incidence of eating disorders is higher in more affluent areas(19) – a trend in contrast with prevalence figures coming from general population samples(39,40) – we observed weak evidence pointing to higher mortality in people living in the most deprived areas compared to people living in more affluent areas. However, confidence intervals were wide and overlapped with those of other estimates. There was also weak evidence of interactions by deprivation for admissions, but although point estimates were higher for people living in more deprived areas, here too confidence intervals overlapped across estimates. Lastly, we also observed weak evidence of potentially increased mortality in ethnic minority compared to white individuals with eating disorders. Although results relating to ethnicity and deprivation were inconclusive, possibly due to our sample being underpowered to detect such differences, it has been shown that people living in more deprived areas and people from ethnic minorities might experience difficulties accessing eating disorder services. A US-based study found that among students with symptoms of eating disorders, those from more deprived socio-economic backgrounds had lower odds of perceiving need for and receiving eating disorder treatment and that people from ethnic minority backgrounds were less likely to have received an eating disorder diagnosis and treatment.(24) People from more disadvantaged socio-economic position might also experience greater barriers in engaging with eating disorder treatment due to direct and indirect costs associated with attending treatment. (41). Future research should further investigate disparities in diagnostic, referral, and treatment patterns by socio-demographic and socio-economic characteristics in UK data as a way to better understand sources and mechanisms underpinning these possible inequalities. Finally, we observed no differences in mortality and admission trends according to calendar year of diagnosis. This finding is worrying as it suggests that despite efforts aimed at expanding access to eating disorder services this has not yet resulted in improvements in patients’ overall health outcomes and leads to calls for improvement in eating disorder treatment.

### Limitations

Our results need to be interpreted in light of some limitations. Despite a sample size of nearly 60,000 patients, mortality analyses might have been underpowered due to the low number of outcome events observed, particularly in diagnosis-specific analyses. Therefore, we repeated mortality analyses on the full primary care dataset. The results of these sensitivity analyses were in line with those of the main analyses but allowed us to detect smaller effects with greater precision. Statistical power considerations also limited our ability to investigate associations with natural and unnatural causes of death (available only in HES-linked data), as the latter were a small proportion of total events.

There are also potential biases associated with the use of electronic health records. For instance, there is potential for misclassification in the exposure, particularly for people with generic eating disorder codes or referral codes – who could be patients on waiting lists for eating disorder services where diagnoses might be confirmed – for whom we could not identify a specific diagnosis. We also found higher rates of cancer-related admissions in patients with anorexia nervosa and EDNOS. Whilst associations with this outcome should be further investigated, we cannot exclude that this pattern might be observed because, in some instances, anorexia nervosa cases included in our dataset could have indicated ‘anorexia’, which is common in patients with cancer. To limit this possibility, in the anorexia nervosa definition, we did not include Read codes that only mentioned ‘anorexia’ and in the EDNOS diagnosis we did not include any codes related to weight loss with organic bases. Nevertheless, we cannot exclude recording errors.

Although cases of eating disorders identified in primary care might be more representative than those seen in secondary care since a minority of cases are referred(19), they are nevertheless a minority of those seen in the general populations(39) and might be a biased sample since access to care might vary across socio-demographic characteristics.(38) We also cannot exclude the potential for Berkson’s bias, which could occur if our exposed population is one with greater physical health problems, hence resulting in both greater likelihood of eating disorders being identified and physical health problems.

A large proportion of the sample had missing data on ethnicity. We took the approach, previously used in other studies, to replace missing ethnicity data with white ethnicity. However, due to small sample size, we were unable to break down ethnicity into more specific categories and explore group-specific associations. This could help inform future guidelines and policies and should be explored in the future in datasets with larger sample sizes.

## Conclusions

Eating disorders are severe psychiatric conditions, which are marked by high mortality rates and frequent hospital admissions. Although mortality was highest for anorexia nervosa, we observed elevated mortality and admissions across the full spectrum of threshold and sub-threshold eating disorder diagnoses. We also observed markedly worse outcomes for specific groups, particularly males. Future research should investigate the mechanisms underlying these inequalities in outcomes, including co-existing physical conditions and the predominant diagnoses which account for the deaths. This would help to target preventative efforts. In the meantime, our findings call not only for improvements in eating disorder treatment (including access to specialist eating disorder services) across the spectrum of diagnoses and socio-demographic groups, but also in the management of eating disorders in primary care. Currently, NICE guidelines only recommend annual physical and mental health checks for people with anorexia nervosa. The extent to which these are currently undertaken should also be investigated, and, given the severity in outcomes we observe, this recommendation should be extended to all eating disorder diagnoses.

## Supplementary Material

Supplemental material

## Figures and Tables

**Table 1:** Sample characteristics. Participants with linked HES data (n=58,735)

		Eating disorder diagnosis						
	Full sample	No eating disorder diagnosis	Any eating disorder diagnosis	Anorexia nervosa	Bulimia nervosa	EDNOS	Eating disorder	Referral to ED clinic
		n(%)	n(%)	n(%)	n(%)	n(%)	n(%)	n(%)
**Sample**	58,735	46,606	12,129	2,513	2,186	3,542	2,851	1,037
**Data source**								
*CPRD Aurum*	36,174 (61.6%)	28,672 (61.5%)	7,502 (61.9%)	1,527 (60.8%)	1,352 (61.8%)	2,531 (71.5%)	1,526 (53.5%)	566 (54.6%)
*CPRD Gold*	22,561 (38.4%)	17,934 (38.5%)	4,627 (38.1%)	986 (39.2%)	834 (38.2%)	1,011 (28.5%)	1,325 (46.5%)	471 (45.4%)
**Diagnosis**								
*Anorexia nervosa*	2,513 (20.8%)	-	2,513 (20.8%)	-	-	-	-	-
*Bulimia nervosa*	2,186 (18.0%)	-	2,186 (18.0%)	-	-	-	-	-
*EDNOS*	3,542 (29.2%)	-	3,542 (29.2%)	-	-	-	-	-
*Eating disorder*	2,851 (23.5%)	-	2,851 (23.5%)	-	-	-	-	-
*Referral to ED clinic*	1,037 (8.5%)	-	1,037 (8.5%)	-	-	-	-	-
**Gender**								
*Male*	5,786 (9.9%)	4,594 (9.9%)	1,192 (9.8%)	157 (6.2%)	123 (5.3%)	437 (12.3%)	364 (12.8%)	111 (10.7%)
*Female*	52,949 (90.1%)	42,012 (90.1%)	10,937(90.2%)	2,356 (93.8%)	2,063 (94.4%)	3,105 (87.7%)	2,487 (87.2%)	926 (89.3%)
**Ethnicity**								
*Asian*	2,816 (4.8%)	2,504 (5.4%)	312 (2.6%)	56 (2.2%)	58 (2.7%)	113 (3.2%)	66 (2.3%)	19 (1.8%)
*Black*	1,109 (1.9%)	958 (2.1%)	151 (1.2%)	24 (1.0%)	16 (0.7%)	49 (1.4%)	43 (1.5%)	19 (1.8%)
*White*	53,819 (91.6%)	42,339 (90.8%)	11,480 (94.6%)	2,410 (95.9%)	2,081 (95.2%)	3,319 (93.7%)	2,692 (94.4%)	978 (94.3%)
*Mixed*	541 (0.9%)	425 (0.9%)	116 (1.0%)	14 (0.6%)	17 (0.8%)	41 (1.16%)	32 (1.1%)	12 (1.2%)
*Other*	450 (0.8%)	380 (0.8%)	70 (0.6%)	9 (0.4%)	14 (0.6%)	20 (0.6%)	18 (0.6%)	9 (0.9%)
**Age (years)**								
*11 - 20*	20,166 (43.3%)	20,166 (43.3%)	5,232 (43.1%)	1,466 (58.3%)	693 (31.7%)	1,325 (38.2%)	1,319 (46.3%)	402 (38.8%)
*21 - 30*	14,954 (32.1%)	14,954 (32.1%)	3,908 (32.2%)	620 (24.7%)	905 (41.4%)	1,183 (33.4%)	860 (30.2%)	340 (32.8%)
*31 - 40*	6,625 (14.2%)	6,625 (14.2%)	1,730 (14.3%)	231 (9.2%)	406 (18.6%)	544 (15.4%)	395 (13.9%)	154 (14.9%)
*41- 50*	3,445 (7.4%	3,445 (7.4%	895 (7.4%)	132 (5.3%)	145 (6.6%)	312 (8.8%)	196 (6.9%)	110 (10.6%)
*51 - 60*	1,416 (3.0%)	1,416 (3.0%)	364 (3.0%)	64 (2.5%)	37 (1.7%)	151 (4.3%)	81 (2.8%)	31 (3.0%)
**Fifth of area-level** **deprivation**								
*1*^*st*^ *(least deprived)*	10,756 (23.1%)	10,756 (23.1%)	2,646 (21.8%)	610 (24.3%)	450 (20.6%)	801 (22.6%)	566 (19.9%)	219 (21.1%)
*2*^*nd*^	10,269 (22.0%)	10,269 (22.0%)	2,708 (22.3%)	603 (24.0%)	507 (23.2%)	745 (21.0%)	645 (22.6%)	208 (20.1%)
*3*^*rd*^	9,015 (19.3%)	9,015 (19.3%)	2,308 (19.0%)	492 (19.6%)	405 (18.5%)	675 (19.1%)	529 (18.6%)	207 (20.0%)
*4*^*th*^	9,514 (20.4%)	9,514 (20.4%)	2,546 (21.0%)	467 (18.6%)	495 (22.6%)	790 (22.3%)	588 (20.6%)	206 (19.9%)
*5*^*th*^ *(most deprived)*	7,052 (15.1%)	7,052 (15.1%)	1,921 (15.8%)	341 (13.6%)	329 (15.1%)	531 (15.0%)	523 (18.3%)	197 (19.0%)
***Region***								
*East England*	4,918 (8.4%)	3,916 (8.4%)	1,002 (8.3%)	174 (6.9%)	175 (8.0%)	293 (8.3%)	277 (9.7%)	83 (8.0%)
*East Midlands*	3,427 (5.8%)	2,736 (5.9%)	691 (5.7%)	117 (4.7%)	143 (6.5%)	248 (7.0%)	145 (5.1%)	38 (3.7%)
*London*	7,451 (12.7%)	5,907 (12.7%)	1,544 (12.7%)	330 (13.1%)	302 (13.8%)	412 (11.6%)	355 (12.5%)	145 (14.0%)
*North East*	692 (1.2%)	549 (1.2%)	143 (1.2%)	29 (1.2%)	28 (1.3%)	37 (1.0%)	37 (1.3%)	12 (1.2%)
*North West*	6,523 (11.1%)	5,173 (11.1%)	1,350 (11.1%)	248 (9.9%)	228 (10.4%)	360 (10.2%)	388 (13.6%)	129 (12.4%)
*South Central*	10,248 (17.4%)	8,128 (17.4%)	2,120 (17.5%)	491 (19.5%)	385 (17.6%)	589 (16.6%)	491 (17.2%)	164 (15.8%)
*South East Coast*	5,663 (9.6%)	4,489 (9.6%)	1,174 (9.7%)	246 (9.8%)	196 (9.0%)	355 (10.0%)	264 (9.3%)	113 (10.9%)
*South West*	10,153 (17.3%)	8,025 (17.2%)	2,128 (17.5%)	506 (20.1%)	384 (17.6%)	592 (16.7%)	484 (17.0%)	162 (15.6%)
*West Midlands*	5,882 (10.0%)	4,673 (10.0%)	1,209 (10.0%)	239 (9.5%)	192 (8.8%)	338 (9.5%)	307 (10.8%)	133 (12.8%)
*Yorkshire*	3,778 (6.4%)	3,010 (6.4%)	768 (6.3%)	133 (5.3%)	156 (7.1%)	318 (9.0%)	103 (3.6%)	58 (5.6%)

**Table 2:** Univariable and multivariable Cox regression analyses for the association between eating disorder diagnoses and all-cause mortality. Main analyses based on the sample of patients with linked CPRD-HES data and complete IMD data (n = 58,735) and sensitivity analyses based on the full non-linked CPRD sample (n=167,630)

	Eating disorder diagnosis						
	No eating disorder	Any eating disorder	Anorexia Nervosa	Bulimia Nervosa	Generic eating disorder code	EDNOS	Referral only
**Main analyses in linked sample (n=58,735)**							
Person-years follow-up	235,467.80	70,158.86	14868.16	12770.88	16,565.24	20,739.91	5,214.68
**All-cause mortality**							
***Deaths (n)***	213	138	40	<15	31	47	<10
	***HR (95% CI)***	***HR (95% CI)***	***HR (95% CI)***	***HR (95% CI)***	***HR (95% CI)***	***HR (95% CI)***	***HR (95% CI)***
*Univariable model*	Ref	2.15 (1.73-2.66)	2.91 (2.07-4.07)	1.19 (0.69-2.04)	2.05 (1.41-2.99)	2.45 (1.79-3.37)	1.35 (0.60-3.04)
*Multivariable model*	Ref	2.15 (1.73-2.67)	3.49 (2.43-5.01)	1.20 (0.84-2.08)	2.14 (1.47-3.12)	2.11 (1.54-2.90)	1.27 (0.56-2.86)

**Sensitivity analyses of *all-cause mortality* in the full non-linked CPRD dataset (n=167,630)**							
	**No eating disorder**	**Any eating disorder**	**Anorexia Nervosa**	**Bulimia Nervosa**	**Generic eating disorder code**	**EDNOS**	**Referral only**
Person-years follow-up	723098.90	204846.20	41134.13	39370.78	48411.87	58901.33	17028.08
***Deaths (n)***	412	692	105	50	92	141	24
	***HR (95% CI)***	***HR (95% CI)***	***HR (95% CI)***	***HR (95% CI)***	***HR (95% CI)***	***HR (95% CI)***	***HR (95% CI)***
*Univariable model*	Ref	2.08 (1.84-2.35)	2.56 (2.09-3.15)	1.31 (0.98-1.75)	1.98 (1.59-2.46)	2.45 (2.04-2.94)	1.58 (1.05-2.38)
*Multivariable model*	Ref	2.17 (1.92-2.45)	3.40 (2.73-4.23)	1.42 (1.05-1.91)	2.16 (1.74-2.69)	2.14 (1.78-2.56)	1.57 (1.05-2.37)

List of abbreviation: HR = hazard ratios, CI = confidence intervals Multivariable model adjusted for: gender, ethnicity, age, calendar year, IMD. In sensitivity analyses IMD was not available across all four UK countries and was thus not adjusted for.

**Table 3:** Stratified analyses for the association between any eating disorder diagnosis and all-cause mortality. P-values presented refer to those for the interaction between exposure (any eating disorder diagnosis vs no eating disorder) and socio-demographic characteristics fitted in the multivariable Cox regression analyses of all-cause mortality presented in Table 2. Analyses based on the main analytical sample (n=58,735) as well as the full not-linked CPRD dataset used for sensitivity analyses (n=167,630).

	Main analyses of all-cause mortality n=58,735		Sensitivity analyses of all-cause mortality n=167,630	
	Multivariable model Hazard Ratio (95% CI)	p-value for interaction	Multivariable model Hazard Ratio (95% CI)	p-value for interaction
Gender*diagnosis	-	-	-	**-**
*Females*	1.85 (1.45-2.35)	0.003	1.80 (1.56-2.07)	<0.0001
*Males*	4.60 (2.74-7.73)		4.36 (3.29-5.78)	
Ethnicity*diagnosis				
*White*	2.10 (1.69-2.62)	0.09	2.13 (1.88-2.42)	0.09
*Ethnic minority*	3.91 (1.30-11.75)		3.35 (1.45-7.73)	
Age*diagnosis				
*11-20*	1.67 (0.88-3.18)		2.27 (1.58-3.25)	
*21-30*	2.07 (1.21-3.51)		2.18 (1.58-2.99)	
*31-40*	1.75 (1.06-2.88)	0.10	1.96 (1.46-2.64)	0.98
*41-50*	2.20 (1.44-3.34)		2.10 (1.64-2.69)	
*51-60*	2.97 (1.95-4.51)		2.35 (1.83-3.00)	
IMD*diagnosis				
*1*^*st*^ *(least deprived)*	2.12 (1.23-3.64)		-	
*2*^*nd*^	1.24 (0.70-2.20)		-	
*3*^*rd*^	2.38 (1.43-3.97)	0.05	-	
*4*^*th*^	2.05 (1.35-3.09)		-	
*5*^*th*^ *(most deprived)*	3.17 (2.02-4.99)		-	
Year*diagnosis	-	0.56	-	0.41

List of abbreviations: CI= confidence interval IMD was not available across all four countries in the sample used for sensitivity analyses, hence it was not used.

**Table 4:** Univariable and multivariable Poisson regression analyses for the association between eating disorder diagnoses and admissions for physical health problems. Sample of participants with linked CPRD-HES data and complete IMD data (n= 58,735).

	No diagnosis	Any eating disorder	Anorexia nervosa	Bulimia nervosa	Eating disorder	EDNOS	Referral to eating disorder service
Person-years follow-up	235,467.80	70,158.86	14,868.16	12,770.88	16,565.24	20,739.91	5,214.68
**Any admissions (physical health)**							
*Admissions (n)*	13,118	7,697	1,705	1,299	1,448	2,714	531
*Incidence per 1,000*	55.71	109.71	114.67	101.72	87.41	130.86	101.83
	***IRR (95% CI)***	***IRR (95% CI)***	***IRR (95% CI)***	***IRR (95% CI)***	***IRR (95% CI)***	***IRR (95% CI)***	***IRR (95% CI)***
*Univariable model*	Ref	1.97 (1.91-2.03)	2.06 (1.96-2.16)	1.83 (1.72-1.93)	1.57 (1.49-1.66)	2.35 (2.25-2.45)	1.83 (1.67-1.99)
*Multivariable model*	Ref	1.99 (1.94-2.05)	2.28 (2.17-2.40)	1.81 (1.71-1.92)	1.62 (1.54-1.71)	2.27 (2.18-2.37)	1.71 (1.57-1.87)
**Planned (physical health)**							
*Admissions (n)*	8,496	4,490	850	833	797	1,699	311
*Incidence per 1,000*	36.08	64.00	57.17	65.23	48.11	81.92	59.64
	***IRR (95% CI)***	***IRR (95% CI)***	***IRR (95% CI)***	***IRR (95% CI)***	***IRR (95% CI)***	***IRR (95% CI)***	***IRR (95% CI)***
*Univariable model*	Ref	1.77 (1.71-1.83)	1.58 (1.48-1.70)	1.81 (1.68-1.94)	1.33 (1.24-1.43)	2.27 (2.15-2.39)	1.65 (1.47-1.85)
*Multivariable model*	Ref	1.80 (1.74-1.87)	1.79 (1.67-1.92)	1.79 (1.66-1.92)	1.40 (1.30-1.50)	2.16 (2.05-2.23)	1.57 (1.40-1.76)
**Emergency (physical health)**							
*Admissions (n)*	4,622	3,207	855	466	651	1,015	220
*Incidence per 1,000*	19.63	45.71	57.51	36.49	39.30	48.94	42.19
	***IRR (95% CI)***	***IRR (95% CI)***	***IRR (95% CI)***	***IRR (95% CI)***	***IRR (95% CI)***	***IRR (95% CI)***	***IRR (95% CI)***
*Univariable model*	Ref	2.33 (2.27-2.44)	2.93 (2.72-3.15)	1.86 (1.69-2.04)	2.00 (1.84-2.17)	2.49 (2.33-2.67)	2.15 (1.87-2.45)
*Multivariable model*	Ref	2.35 (2.25-2.46)	3.11 (2.89-3.34)	1.88 (1.71-2.07)	2.02 (1.86-2.19)	2.49 (2.32-2.66)	1.98 (1.72-2.26)
**Emergency accidents, injuries and substance misuse**							
*Admissions (n)*	1,272	741	330	238	270	337	97
*Incidence per 1,000*	3.15	18.13	22.19	18.64	16.30	16.25	18.60
	***IRR (95% CI)***	***IRR (95% CI)***	***IRR (95% CI)***	***IRR (95% CI)***	***IRR (95% CI)***	***IRR (95% CI)***	***IRR (95% CI)***
*Univariable model*	Ref	5.76 (5.26-6.31)	7.05 (6.19-8.02)	5.92 (5.11-6.84)	5.18 (4.50-5.95)	5.16 (4.53-5.87)	5.18 (4.75-7.26)
*Multivariable model*	Ref	5.26 (5.24-6.29)	7.07 (6.20-8.05)	6.42 (5.53-7.42)	4.95 (4.30-5.69)	5.24 (4.60-5.96)	5.12 (4.12-6.31)

List of abbreviations: IRR = incidence rate ratio, CI= confidence interval Multivariable model adjusted for: sex, ethnicity, age, calendar year, IMD.

**Table 5:** Stratified analyses for the association between any eating disorder diagnosis and admissions for physical health problems. P-values presented refer to those for the interaction between exposure (any eating disorder diagnosis vs no eating disorder) and socio-demographic characteristics fitted in the multivariable Poisson regression analyses of all-cause mortality presented in Table 4. Analyses based on the main analytical sample (n=58,735).

	Multivariable model Incidence Rate Ratio (95% CI)	p-value for interaction
Gender*diagnosis		
*Females*	1.97 (1.91-2.33)	0.01
*Males*	2.28 (2.07-2.51)	
Ethnicity*diagnosis		
*White*	1.99 (1.93-2.05)	0.42
*Ethnic minority*	2.00 (1.79-2.23)	
Age*diagnosis		
* 11-20*	2.03 (1.92-2.13)	
*21-30*	1.83 (1.73-1.94)	
*31-40*	2.39 (2.24-2.55)	0.36
*41-50*	2.03 (1.89-2.18)	
*51-60*	1.63 (1.48-1.80)	
IMD*diagnosis		
*1*^*st*^ *(least deprived)*	1.96 (1.85-2.09)	
*2*_*nd*_	1.81 (1.72-1.94)	
*3*^*rd*^	2.12 (1.99-2.26)	0.06
*4*^*th*^	2.02 (1.90-2.15)	
*5*^*th*^ *(most deprived)*	2.09 (1.96-2.23)	
Year*diagnosis	-	0.81

List of abbreviations: CI= confidence interval

## Data Availability

The data used in this study are not publicly available as electronic health records are considered sensitive data in the UK. As such and in line with the Data Protection Act, they cannot be shared in open access repositories due to information governance restrictions in place to protect patient confidentiality. CPRD and HES data can be accessed once approval has been obtained through an application to the Clinical Practice Research Datalink.

## References

[1] Arcelus J, Mitchell AJ, Wales J, Nielsen S (2011). Mortality Rates in Patients With Anorexia Nervosa and Other Eating Disorders: A Meta-analysis of 36 Studies. Archives of General Psychiatry.

[2] Iwajomo T, Bondy SJ, de Oliveira C, Colton P, Trottier K, Kurdyak P (2021). Excess mortality associated with eating disorders: population-based cohort study. Br J Psychiatry.

[3] Kask J, Ekselius L, Brandt L, Kollia N, Ekbom A, Papadopoulos FC (2016). Mortality in Women With Anorexia Nervosa: The Role of Comorbid Psychiatric Disorders. Psychosom Med.

[4] Kask J, Ramklint M, Kolia N, Panagiotakos D, Ekbom A, Ekselius L (2017). Anorexia nervosa in males: excess mortality and psychiatric co-morbidity in 609 Swedish in-patients. Psychol Med.

[5] Tseng MCM, Chien LN, Tu CY, Liu HY (2023). Mortality in anorexia nervosa and bulimia nervosa: A population-based cohort study in Taiwan, 2002–2017. International Journal of Eating Disorders.

[6] Franko DL, Keshaviah A, Eddy KT, Krishna M, Davis MC, Keel PK (2013). A longitudinal investigation of mortality in anorexia nervosa and bulimia nervosa. Am J Psychiatry.

[7] Fichter MM, Quadflieg N (2016). Mortality in eating disorders - results of a large prospective clinical longitudinal study. Int J Eat Disord.

[8] Himmerich H, Hotopf M, Shetty H, Schmidt U, Treasure J, Hayes RD (2019). Psychiatric comorbidity as a risk factor for mortality in people with anorexia nervosa. Eur Arch Psychiatry Clin Neurosci.

[9] Zerwas S, Larsen JT, Petersen L, Thornton LM, Mortensen PB, Bulik CM (2015). The Incidence of Eating Disorders in a Danish Nationwide Register Study Associations with Suicide Risk and Mortality. J Psychiatr Res.

[10] Demmler JC, Brophy ST, Marchant A, John A, Tan JOA (2020). Shining the light on eating disorders, incidence, prognosis and profiling of patients in primary and secondary care: national data linkage study. Br J Psychiatry.

[11] John A, Marchant A, Demmler J, Tan J, DelPozo-Banos M (2021). Clinical management and mortality risk in those with eating disorders and self-harm: e-cohort study using the SAIL databank. BJPsych Open.

[12] Huas C, Godart N, Caille A, Pham-Scottez A, Foulon C, Divac SM (2013). Mortality and its predictors in severe bulimia nervosa patients. Eur Eat Disord Rev.

[13] Hoang U, Goldacre M, James A (2014). Mortality following hospital discharge with a diagnosis of eating disorder: national record linkage study, England, 2001-2009. Int J Eat Disord.

[14] Striegel-Moore RH, DeBar L, Wilson GT, Dickerson J, Rosselli F, Perrin N (2008). Health services use in eating disorders. Psychological Medicine.

[15] Couturier J, Gayowsky A, Findlay S, Webb C, Sami S, Chan AKC (2022). A retrospective cohort study examining health care utilization patterns in individuals diagnosed with an eating disorder in childhood and/or adolescence. International Journal of Eating Disorders.

[16] Tith RM, Paradis G, Potter BJ, Low N, Healy-Profitós J, He S (2020). Association of Bulimia Nervosa With Long-term Risk of Cardiovascular Disease and Mortality Among Women. JAMA Psychiatry.

[17] Watson HJ, Jangmo A, Smith T, Thornton LM, von Hausswolff-Juhlin Y, Madhoo M (2018). A register-based case-control study of health care utilization and costs in binge-eating disorder. J Psychosom Res.

[18] Tseng MCM, Tu CY, Chang YT (2021). Healthcare use and costs of adults with anorexia nervosa and bulimia nervosa in Taiwan. Int J Eat Disord.

[19] Wood S, Marchant A, Allsopp M, Wilkinson K, Bethel J, Jones H (2019). Epidemiology of eating disorders in primary care in children and young people: a Clinical Practice Research Datalink study in England. BMJ Open.

[20] Eseaton P, Sanwo E, Anighoro SO, John E, Okobia NO, Enosolease U (2022). Emergency Department Utilization by Patients With Eating Disorders: A National Population-Based Study. Cureus.

[21] de Oliveira C, Colton P, Cheng J, Olmsted M, Kurdyak P (2017). The direct health care costs of eating disorders among hospitalized patients: A population-based study. International Journal of Eating Disorders.

[22] Ahmed M, Maguire S, Dann KM, Scheneuer F, Kim M, Miskovic-Wheatley J (2024). Socioeconomic inequity in the utilization of healthcare among people with eating disorders in Australia. Psychol Med.

[23] Sangha S, Oliffe JL, Kelly MT, McCuaig F (2019). Eating Disorders in Males: How Primary Care Providers Can Improve Recognition, Diagnosis, and Treatment. Am J Mens Health.

[24] Sonneville KR, Lipson SK (2018). Disparities in eating disorder diagnosis and treatment according to weight status, race/ethnicity, socioeconomic background, and sex among college students. Int J Eat Disord.

[25] Herrett E, Gallagher AM, Bhaskaran K, Forbes H, Mathur R, van Staa T (2015). Data Resource Profile: Clinical Practice Research Datalink (CPRD). International Journal of Epidemiology.

[26] Launders N, Kirsh L, Osborn DPJ, Hayes JF (2022). The temporal relationship between severe mental illness diagnosis and chronic physical comorbidity: a UK primary care cohort study of disease burden over 10 years. The Lancet Psychiatry.

[27] Launders N, Hayes JF, Price G, Osborn DP (2022). Clustering of physical health multimorbidity in people with severe mental illness: An accumulated prevalence analysis of United Kingdom primary care data. PLOS Medicine.

[28] Cucchi A, Ryan D, Konstantakopoulos G, Stroumpa S, Kaçar AŞ, Renshaw S (2016). Lifetime prevalence of non-suicidal self-injury in patients with eating disorders: a systematic review and meta-analysis. Psychological Medicine.

[29] Pisetsky EM, Thornton LM, Lichtenstein P, Pedersen NL, Bulik CM (2013). Suicide Attempts in Women with Eating Disorders. Journal of abnormal psychology.

[30] Warne N, Heron J, Mars B, Moran P, Stewart A, Munafò M (2021). Comorbidity of self-harm and disordered eating in young people: Evidence from a UK population-based cohort. Journal of Affective Disorders.

[31] Bahji A, Mazhar MN, Hudson CC, Nadkarni P, MacNeil BA, Hawken E (2019). Prevalence of substance use disorder comorbidity among individuals with eating disorders: A systematic review and meta-analysis. Psychiatry Research.

[32] Haines MS (2023). Endocrine complications of anorexia nervosa. J Eat Disord.

[33] Milano W, Capasso A (2018). Neuroendocrine and Metabolic Disorders in Bulimia Nervosa. Endocr Metab Immune Disord Drug Targets.

[34] Schorr M, Miller KK (2016). The endocrine manifestations of anorexia nervosa: mechanisms and management. Nature reviews Endocrinology.

[35] Puckett L (2023). Renal and electrolyte complications in eating disorders: a comprehensive review. J Eat Disord.

[36] Santonicola A, Gagliardi M, Guarino MPL, Siniscalchi M, Ciacci C, Iovino P (2019). Eating Disorders and Gastrointestinal Diseases. Nutrients.

[37] Suszko M, Sobocki J, Imieliński C (2022). Mortality in extremely low BMI anorexia nervosa patients - implications of gastrointestinal and endocrine system dysfunction. Psychiatr Pol.

[38] Catalá-López F, Forés-Martos J, Driver JA, Page MJ, Hutton B, Ridao M (2019). Association of Anorexia Nervosa With Risk of Cancer: A Systematic Review and Meta-analysis. JAMA Network Open.

[39] Solmi F, Hatch SL, Hotopf M, Treasure J, Micali N (2014). Prevalence and correlates of disordered eating in a general population sample: The South East London Community Health (SELCoH) study. Social Psychiatry and Psychiatric Epidemiology.

[40] NHS England Digital Health Survey for England 2019 [NS].

[41] Bailey-Straebler S, Glasofer DR, Ojeda J, Attia E (2024). Equitable access to evidence-based treatment for eating disorders for patients with low-income: identifying barriers and exploring solutions. the Cognitive Behaviour Therapist.

