# Supplementary material for: Mortality and hospital admissions in people with eating disorders. A longitudinal cohort study in secondary-care linked English primary care records
Source: Br J Psychiatry. Author manuscript; Available in PMC 2026 Jul 1. (PMC7617822; doi:10.1192/bjp.2025.69)
Supplement: Supplemental material [file EMS203447-supplement-Supplemental_material.docx]

**Mortality and hospital admissions in people with eating disorders. A longitudinal cohort study in secondary-care linked English primary care records.**

*Annie Jeffery^1^ PhD, Joseph F. Hayes^1,2^ PhD, Naomi Launders^1^ PhD, Glyn Lewis^1,2^ PhD, David Osborn^1,2^ PhD, Helen Bould^3, 4,5^ PhD, Naomi Warne^3^ PhD, Francesca Solmi^1^ PhD*

^1^ Division of Psychiatry, UCL, London, UK

^2^ North London Mental Health Partnership, London, UK

^3^ Centre for Academic Mental Health, Population Health Sciences, Bristol Medical School,

University of Bristol

^4^ Gloucestershire Health and Care NHS Foundation Trust, Gloucestershire, United Kingdom

^5^ MRC Integrative Epidemiology Unit, Bristol Medical School, University of Bristol, United Kingdom

**Corresponding author**

Professor Francesca Solmi

UCL Division of Psychiatry,

Maple House, Wing A, 6^th^ Floor

149 Tottenham Court road, W1T 7NF,

London, UK.

Phone: 02076799643 (ext: 09643)

Email: [Francesca.solmi@ucl.ac.uk](mailto:Francesca.solmi@ucl.ac.uk)

Table of Contents

[Supplemental method 1: Study dataset 3](#_Toc182467429)

[Supplemental method 2: Definitions of eating disorder diagnoses 3](#_Toc182467430)

[Supplemental method 3: List of eating disorder Read Codes 4](#_Toc182467431)

[Supplemental method 4: ICD10 Codes used for hospital admissions in linked HES data 6](#_Toc182467432)

[Supplemental method 5: Index of Multiple deprivation 6](#_Toc182467433)

[Supplemental method 6: STROBE Statement—checklist of items that should be included in reports of observational studies 7](#_Toc182467434)

[Supplemental Table 1: Causes of hospital admissions 9](#_Toc182467435)

[References 10](#_Toc182467436)

## Supplemental method 1: Study dataset

The CPRD is a longitudinal dataset of electronic primary care records for over 60 million people (of whom 18 million are currently registered and have data spanning over 20 years), across 2000 primary care practices in the UK using EMIS or Vision electronic healthcare records software. CPRD has been shown to be representative of the UK population.(1,2) In this study, we included primary care patients with linked HES data. Specifically, we used the HES Admitted Patient Care dataset, containing details of all admissions to English NHS healthcare providers. This includes admission and discharge dates, admission method (such as planned, or emergency), and diagnoses (using ICD-10 codes). CPRD practices can opt out of HES linkage, and in those where linkage is available, patients are deemed eligible if they have enough data to be linked. We selected participants who were eligible for linkage based on either criterion. Albeit in a different sample, previous research (3) has shown that patients with and without HES linkage are comparable in terms of socio-demographic characteristics, BMI, and recorded risk behaviours such as smoking or alcohol use.

## Supplemental method 2: Definitions of eating disorder diagnoses

Records of eating disorder diagnoses were derived from primary care Read codes and included the following categories: anorexia nervosa, bulimia nervosa, and EDNOS. Diagnostic transition, particularly from threshold to sub-threshold eating disorder presentations, is common.(4) In such cases, we took a hierarchical approach to attributing the exposure by giving a diagnosis of anorexia nervosa to anyone ever diagnosed with anorexia nervosa; a diagnosis of bulimia nervosa to anyone who was ever diagnosed with bulimia nervosa, but never with anorexia nervosa; and a diagnosis of EDNOS to anyone diagnosed with EDNOS but never with anorexia nervosa or bulimia nervosa. Date of eating disorder onset was maintained as the date in which the first eating disorder Read code ever appeared in a person’s records.

In our analytical sample (n=58,753):

- 5.7%, 14.2%, and 24.4% of patients with anorexia nervosa had a lifetime diagnosis of bulimia nervosa, EDNOS, and a generic eating disorder code, respectively.
- 19.3%, 18.4% of patients with bulimia nervosa had a lifetime diagnosis of EDNOS, and a generic eating disorder code, respectively
- 10.8% of patients with EDNOS had a lifetime generic eating disorder code.

Clinical referral codes where present in 11.2% of patients with EDNOS, 16.9% of patients with bulimia nervosa, and 22.6% of patients with anorexia nervosa.

## Supplemental method 3: List of eating disorder Read Codes

| **description** | **medcode** | **ed_type** | **database** |
| --- | --- | --- | --- |
| H/O: anorexia nervosa | 251631011 | Anorexia nervosa | aurum |
| Binge eating | 370419010 | EDNOS | aurum |
| Emotional eating | 2318751000000116 | EDNOS | aurum |
| Suspected binge eating disorder | 2308361000000115 | EDNOS | aurum |
| Referral to eating disorders clinic | 1780181019 | reffered_to_ed_clinic | aurum |
| Seen in eating disorder clinic | 664031000000110 | reffered_to_ed_clinic | aurum |
| Anorexia nervosa | 94597012 | Anorexia nervosa | aurum |
| Other and unspecified non-organic eating disorders | 295436010 | EDNOS | aurum |
| Unspecified non-organic eating disorder | 295437018 | EDNOS | aurum |
| Bulimia (non-organic overeating) | 527421000006116 | Bulimia nervosa | aurum |
| Compulsive eating disorder | 577901000006119 | EDNOS | aurum |
| Psychogenic vomiting NOS | 295442014 | EDNOS | aurum |
| Non-organic loss of appetite | 295444010 | EDNOS | aurum |
| Other specified non-organic eating disorder | 295451018 | EDNOS | aurum |
| Non-organic eating disorder NOS | 295452013 | EDNOS | aurum |
| [X]Eating disorders | 296361019 | generic_ed_code | aurum |
| [X]Anorexia nervosa | 363321000006111 | Anorexia nervosa | aurum |
| [X]Atypical anorexia nervosa | 366521000006113 | EDNOS | aurum |
| [X]Bulimia nervosa | 368051000006111 | Bulimia nervosa | aurum |
| [X]Bulimia NOS | 368061000006113 | EDNOS | aurum |
| [X]Hyperorexia nervosa | 389311000006116 | EDNOS | aurum |
| [X]Atypical bulimia nervosa | 366541000006118 | EDNOS | aurum |
| [X]Overeating associated with other psychological disturbncs | 417851000006113 | EDNOS | aurum |
| [X]Psychogenic overeating | 424061000006117 | EDNOS | aurum |
| [X]Vomiting associated with other psychological disturbances | 432551000006118 | EDNOS | aurum |
| [X]Psychogenic vomiting | 424151000006115 | EDNOS | aurum |
| [X]Other eating disorders | 401889011 | EDNOS | aurum |
| [X]Psychogenic loss of appetite | 424051000006119 | EDNOS | aurum |
| [X]Eating disorder, unspecified | 296379015 | EDNOS | aurum |
| [D]Anorexia NOS | 317235018 | EDNOS | aurum |
| [D]Bulimia NOS | 1222496015 | EDNOS | aurum |
| Anorexia nervosa | 2135 | Anorexia nervosa | gold |
| Attempts to counteract effects of bingeing | 38949 | Bulimia nervosa | gold |
| Binge eating | 26518 | EDNOS | gold |
| Bulimia (non-organic overeating) | 4377 | Bulimia nervosa | gold |
| Compulsive eating disorder | 11608 | EDNOS | gold |
| Emotional eating | 108635 | EDNOS | gold |
| H/O: anorexia nervosa | 8027 | Anorexia nervosa | gold |
| Non-organic eating disorder NOS | 32892 | EDNOS | gold |
| Non-organic loss of appetite | 22820 | EDNOS | gold |
| Other and unspecified non-organic eating disorders | 7743 | EDNOS | gold |
| Other specified non-organic eating disorder | 61236 | EDNOS | gold |
| Psychogenic vomiting NOS | 3422 | EDNOS | gold |
| Referral to eating disorders clinic | 11612 | reffered_to_ed_clinic | gold |
| Repeated self-induced vomiting | 51370 | Bulimia nervosa | gold |
| Seen in eating disorder clinic | 95883 | reffered_to_ed_clinic | gold |
| Self-induced vomiting to lose weight | 52605 | Bulimia nervosa | gold |
| Suspected binge eating disorder | 108164 | EDNOS | gold |
| Unspecified non-organic eating disorder | 44544 | EDNOS | gold |
| [D]Anorexia NOS | 53746 | EDNOS | gold |
| [D]Bulimia NOS | 605 | EDNOS | gold |
| [X]Anorexia nervosa | 30570 | Anorexia nervosa | gold |
| [X]Atypical anorexia nervosa | 34929 | EDNOS | gold |
| [X]Atypical bulimia nervosa | 33863 | EDNOS | gold |
| [X]Bulimia NOS | 6583 | EDNOS | gold |
| [X]Bulimia nervosa | 9581 | Bulimia nervosa | gold |
| [X]Eating disorder, unspecified | 36946 | EDNOS | gold |
| [X]Eating disorders | 6159 | generic_ed_code | gold |
| [X]Hyperorexia nervosa | 96475 | EDNOS | gold |
| [X]Other eating disorders | 34995 | EDNOS | gold |
| [X]Overeating associated with other psychological disturbncs | 39383 | EDNOS | gold |
| [X]Psychogenic loss of appetite | 17203 | EDNOS | gold |
| [X]Psychogenic overeating | 17439 | EDNOS | gold |
| [X]Psychogenic vomiting | 6796 | EDNOS | gold |
| [X]Vomiting associated with other psychological disturbances | 16622 | EDNOS | gold |

## Supplemental method 4: ICD10 Codes used for hospital admissions in linked HES data

We included only epitype = 1 (general episode) .

Planned admissions included admimeth = 11, 12, 13.

Emergency admissions included admimeth = 21, 22, 23, 24, 25, 28, 2A, 2D.

Admissions for physical health included primary diagnosis with ICD codes starting with: A, B, C, D0, D1, D2, D3, D4, D5, D6, D7, D8, E, G, H0, H1, H2, H3, H4, H5, H6, H7, H8, H9, I, J, K, L, M, N, R0, R1, R20, R21, R22, R23, R25, R26, R26, R28, R29, R3, R50, R51, R52, R53, R55, R56, R57, R59, R64, R65, R68.0, R70, R71, R72, R73, R74, R75, R76, R77, R79, R80, R81, R82.0, R82.1, R82.2, R82.3, R82.4, R82.6, R82.7, R82.8, R82.9, R83, R84, R85, R86, R87, R88, R89, R90, R91, R92, R93, R94, U0, U1, U2, U3, U4, U8, Z00.0, Z00.1, Z00.2, Z00.3, Z00.6, Z00.7, Z00.8, Z01, Z02, Z03.0, Z03.1, Z03.3, Z03.4, Z03.5, Z03.8, Z03.9, Z08, Z09.1, Z09.2,Z10, Z11, Z12, Z13.0, Z13.1, Z13.2, Z13.5, Z13.6, Z13.8, Z13.9, Z40, Z45, Z49, Z50, Z51.0, Z51.1, Z51.2, Z51.6, Z54.1, Z54.2, Z71.3, Z71.7, Z80, Z85, Z86.0, Z86.1, Z86.2, Z86.3, Z86.6, Z86.7, Z87.0, Z87.1, Z87.2, Z87.3, Z87.4, Z87.6, Z87.7, Z87.8, Z88, Z91.0, Z94, Z95, Z2.

Admissions for accidents/injury included primary diagnosis with ICD codes starting with: R78, S, T0, T10, T11, T12, T13, T14, T15, T16, T17, T18, T19, T2, T30, T31, T32, T33, T34, T35, T36, T37, T38, T39, T4, T5, T60, T61, T62, T63, T64, T65, T66, T67, T68, T69, T70, T71, T72, T73, T74, T75, T76, T77, T78, T79, T9, V, W, X0, X1, X2, X3, X4, X5, X6, X7, X80, X81, X82, X83, X84, X85, X86, X87, X88, X89, X9, Y0, Y1, Y2, Y30, Y31, Y32, Y33, Y34, Y35, Y36, Y85, Y86, Y87, Y89, Y90, Y91, Z03.6, Z04.0, Z04.1, Z04.2, Z04.3, Z04.4, Z04.5, Z04.6, Z04.7, Z04.8, Z04.9, Z09.4, Z50.2, Z50.3, Z54.4, Z71.4, Z71.5, Z71.6, Z72, Z86.4, Z91.5, Z91.6.

## Supplemental method 5: Index of Multiple deprivation

The Index of Multiple Deprivation is the official measure of area-level relative deprivation in England.[1] It includes seven distinct domains of deprivation (income, employment, health deprivation and disability, education, crime, barriers to housing and services, living environment) which are combined and weighted to derive a total score, which in our study we split in fifths along quintiles of distribution.

## Supplemental method 6: STROBE Statement—checklist of items that should be included in reports of observational studies

|  | Item No | Recommendation | Page  No |
| --- | --- | --- | --- |
| **Title and abstract** | 1 | (*a*) Indicate the study’s design with a commonly used term in the title or the abstract | 1 |
|  |  | (*b*) Provide in the abstract an informative and balanced summary of what was done and what was found | 2 |
| Introduction | | | |
| Background/rationale | 2 | Explain the scientific background and rationale for the investigation being reported | 3-4 |
| Objectives | 3 | State specific objectives, including any prespecified hypotheses | 4 |
| Methods | | | |
| Study design | 4 | Present key elements of study design early in the paper | 4 |
| Setting | 5 | Describe the setting, locations, and relevant dates, including periods of recruitment, exposure, follow-up, and data collection | 4-5 |
| Participants | 6 | (*a*) *Cohort study*—Give the eligibility criteria, and the sources and methods of selection of participants. Describe methods of follow-up  *Case-control study*—Give the eligibility criteria, and the sources and methods of case ascertainment and control selection. Give the rationale for the choice of cases and controls  *Cross-sectional study*—Give the eligibility criteria, and the sources and methods of selection of participants | 5 |
|  |  | (*b*) *Cohort study*—For matched studies, give matching criteria and number of exposed and unexposed  *Case-control study*—For matched studies, give matching criteria and the number of controls per case | 5 |
| Variables | 7 | Clearly define all outcomes, exposures, predictors, potential confounders, and effect modifiers. Give diagnostic criteria, if applicable | 5-6, supplement |
| Data sources/ measurement | 8* | For each variable of interest, give sources of data and details of methods of assessment (measurement). Describe comparability of assessment methods if there is more than one group | 5-6, supplement |
| Bias | 9 | Describe any efforts to address potential sources of bias | 7 (sensitivity analyses) |
| Study size | 10 | Explain how the study size was arrived at | 7-8 |
| Quantitative variables | 11 | Explain how quantitative variables were handled in the analyses. If applicable, describe which groupings were chosen and why | 5-6 |
| Statistical methods | 12 | (*a*) Describe all statistical methods, including those used to control for confounding | 6-7 |
|  |  | (*b*) Describe any methods used to examine subgroups and interactions | 7 |
|  |  | (*c*) Explain how missing data were addressed | na |
|  |  | (*d*) *Cohort study*—If applicable, explain how loss to follow-up was addressed  *Case-control study*—If applicable, explain how matching of cases and controls was addressed  *Cross-sectional study*—If applicable, describe analytical methods taking account of sampling strategy | na |
|  |  | (*e*) Describe any sensitivity analyses |  |

Continued on next page

| Results | | | |
| --- | --- | --- | --- |
| Participants | 13* | (a) Report numbers of individuals at each stage of study—eg numbers potentially eligible, examined for eligibility, confirmed eligible, included in the study, completing follow-up, and analysed | 7-8 |
|  |  | (b) Give reasons for non-participation at each stage | na |
|  |  | (c) Consider use of a flow diagram | na |
| Descriptive data | 14* | (a) Give characteristics of study participants (eg demographic, clinical, social) and information on exposures and potential confounders | 8 |
|  |  | (b) Indicate number of participants with missing data for each variable of interest | na |
|  |  | (c) *Cohort study*—Summarise follow-up time (eg, average and total amount) | 22, 24 |
| Outcome data | 15* | *Cohort study*—Report numbers of outcome events or summary measures over time | *22, 24* |
|  |  | *Case-control study—*Report numbers in each exposure category, or summary measures of exposure |  |
|  |  | *Cross-sectional study—*Report numbers of outcome events or summary measures |  |
| Main results | 16 | (*a*) Give unadjusted estimates and, if applicable, confounder-adjusted estimates and their precision (eg, 95% confidence interval). Make clear which confounders were adjusted for and why they were included | 8-10, 22,24 |
|  |  | (*b*) Report category boundaries when continuous variables were categorized | - |
|  |  | (*c*) If relevant, consider translating estimates of relative risk into absolute risk for a meaningful time period | 22, 24 |
| Other analyses | 17 | Report other analyses done—eg analyses of subgroups and interactions, and sensitivity analyses | 8-10, 23,25 |
| Discussion | | | |
| Key results | 18 | Summarise key results with reference to study objectives | 10-11 |
| Limitations | 19 | Discuss limitations of the study, taking into account sources of potential bias or imprecision. Discuss both direction and magnitude of any potential bias | 13-14 |
| Interpretation | 20 | Give a cautious overall interpretation of results considering objectives, limitations, multiplicity of analyses, results from similar studies, and other relevant evidence | 12-13 |
| Generalisability | 21 | Discuss the generalisability (external validity) of the study results | 12-13 |
| Other information | | | |
| Funding | 22 | Give the source of funding and the role of the funders for the present study and, if applicable, for the original study on which the present article is based | 16 |

*Give information separately for cases and controls in case-control studies and, if applicable, for exposed and unexposed groups in cohort and cross-sectional studies.

**Note:** An Explanation and Elaboration article discusses each checklist item and gives methodological background and published examples of transparent reporting. The STROBE checklist is best used in conjunction with this article (freely available on the Web sites of PLoS Medicine at http://www.plosmedicine.org/, Annals of Internal Medicine at http://www.annals.org/, and Epidemiology at http://www.epidem.com/). Information on the STROBE Initiative is available at www.strobe-statement.org.

## Supplemental Table 1: Causes of hospital admissions

|  | | **No eating disorder** | **Any eating disorder** | **Anorexia nervosa** | **Bulimia**  **nervosa** | **Generic eating disorder** | **EDNOS** | **Referral only** |
| --- | --- | --- | --- | --- | --- | --- | --- | --- |
| Infections | 1.13 | 2.47 | 3.03 | 2.04 | 1.93 | 2.75 | 2.49 |  |
| Cancer | 5.47 | 8.41 | 10.69 | 4.54 | 5.86 | 12.58 | 2.88 |  |
| Blood disorders | 1.15 | 2.21 | 3.63 | 3.13 | 0.66 | 2.12 | 1.15 |  |
| Endocrine | 1.50 | 6.80 | 12.04 | 3.21 | 3.80 | 8.05 | 5.18 |  |
| Nervous system | 3.89 | 2.47 | 3.36 | 3.45 | 3.80 | 4.39 | 4.60 |  |
| Eye | 0.78 | 1.88 | 1.55 | 1.17 | 0.78 | 3.62 | 1.15 |  |
| Ear | 0.56 | 0.56 | 0.87 | 0.08 | 0.78 | 0.43 | 0.58 |  |
| CVD | 2.02 | 4.02 | 2.69 | 5.87 | 3.08 | 4.92 | 2.68 |  |
| Respiratory | 3.33 | 6.26 | 6.52 | 5.32 | 6.10 | 6.61 | 6.90 |  |
| Digestive | 11.25 | 21.71 | 21.19 | 18.32 | 19.81 | 27.34 | 15.15 |  |
| Skin conditions | 1.90 | 3.16 | 2.35 | 5.95 | 2.41 | 2.70 | 2.88 |  |
| MSK | 6.30 | 11.20 | 7.80 | 11.51 | 8.99 | 14.51 | 14.00 |  |
| Genitourinary | 7.10 | 13.14 | 13.38 | 14.72 | 9.42 | 14.32 | 15.72 |  |
| Other | 11.92 | 28.35 | 33.16 | 24.23 | 22.46 | 31.58 | 30.11 |  |
| Unnatural | 4.66 | 20.72 | 25.49 | 20.91 | 18.65 | 18.95 | 20.33 |  |

***Rates per 1,000 person-years**

## References

1. Herrett E, Gallagher AM, Bhaskaran K, Forbes H, Mathur R, van Staa T, et al. Data Resource Profile: Clinical Practice Research Datalink (CPRD). International Journal of Epidemiology. 2015 Jun 1;44(3):827–36.

2. Mahadevan P, Harley M, Fordyce S, Hodgson S, Ghosh R, Myles P, et al. Completeness and representativeness of small area socioeconomic data linked with the UK Clinical Practice Research Datalink (CPRD). J Epidemiol Community Health. 2022 Oct 1;76(10):880–6.

3. Launders N, Hayes JF, Price G, Marston L, Osborn DPJ. The incidence rate of planned and emergency physical health hospital admissions in people diagnosed with severe mental illness: a cohort study. Psychological Medicine. 2023 Sep;53(12):5603–14.

4. Schaumberg K, Jangmo A, Thornton LM, Birgegård A, Almqvist C, Norring C, et al. Patterns of diagnostic transition in eating disorders: a longitudinal population study in Sweden. Psychological Medicine. 2019 Apr;49(5):819–27.
